# Highly Selective Anti-Cancer Activity of Cholesterol-Interacting Agents Methyl-β-Cyclodextrin and Ostreolysin A/Pleurotolysin B Protein Complex on Urothelial Cancer Cells

**DOI:** 10.1371/journal.pone.0137878

**Published:** 2015-09-11

**Authors:** Nataša Resnik, Urška Repnik, Mateja Erdani Kreft, Kristina Sepčić, Peter Maček, Boris Turk, Peter Veranič

**Affiliations:** 1 Institute of Cell Biology, Faculty of Medicine, University of Ljubljana, Vrazov trg 2, Ljubljana, Slovenia; 2 Department of Biochemistry and Molecular and Structural Biology, Jožef Stefan Institute, Jamova 39, Ljubljana, Slovenia; 3 Department of Biology, Biotechnical Faculty, University of Ljubljana, Večna pot 111, Ljubljana, Slovenia; Institut du Cerveau et de la Moelle, FRANCE

## Abstract

Cholesterol content can vary distinctly between normal and cancer cells, with elevated levels in cancer cells. Here, we investigated cholesterol sequestration with methyl-β-cyclodextrin (MCD), and pore-formation with the ostreolysin A/pleurotolysin B (OlyA/PlyB) protein complex that binds to cholesterol/sphingomyelin-rich membrane domains. We evaluated the effects on viability of T24 invasive and RT4 noninvasive human urothelial cancer cells and normal porcine urothelial (NPU) cells. Cholesterol content strongly correlated with cancerous transformation, as highest in the T24 high-grade invasive urothelial cancer cells, and lowest in NPU cells. MCD treatment induced prominent cell death of T24 cells, whereas OlyA/PlyB treatment resulted in greatly decreased viability of the RT4 low-grade noninvasive carcinoma cells. Biochemical and transmission electron microscopy analyses revealed that MCD and OlyA/PlyB induce necrotic cell death in these cancer cells, while viability of NPU cells was not significantly affected by either treatment. We conclude that MCD is more toxic for T24 high-grade invasive urothelial cancer cells, and OlyA/PlyB for RT4 low-grade noninvasive urothelial cancer cells, and neither is toxic for NPU cells. The cholesterol and cholesterol/sphingomyelin-rich membrane domains in urothelial cancer cells thus constitute a selective therapeutic target for elimination of urothelial cancer cells.

## Introduction

In most eukaryotic cells, the plasma-membrane cholesterol content represents as much as 90% of total cell cholesterol [1,2]. Cholesterol is a crucial membrane component, and it affects membrane structure and function, including membrane fluidity and membrane protein activity [3,4,5]. Together with sphingomyelin, cholesterol accumulates in membrane domains that are known as membrane rafts. The specific lipid and protein compositions of these cholesterol/ sphingomyelin-rich membrane domains are implicated in many key signaling pathways associated with cell growth, migration and apoptosis [6,7].

Cholesterol metabolism is strictly regulated, to maintain the appropriate cholesterol content in healthy cells. Clinical and experimental evidence suggests that perturbations in cholesterol metabolism can have important roles in cancerogenesis and tumor development (reviewed in [8,9]). Such perturbations have been demonstrated in several malignancies [10,11,12], and cholesterol metabolites can promote or suppress cancers [13]. Increased cholesterol levels have been observed in noninvasive [10,14,15] and invasive [16] cancer cells, and these cholesterol increases can modulate membrane-raft dynamics and raft-related coordination of various signaling pathways in cancer cells [17].

The cholesterol content of cancer cells is usually increased through up-regulation of 3-hydroxy-3-methylglutaryl (HMG)-CoA reductase, a regulatory enzyme in cholesterol synthesis, which leads to coalescence of membrane rafts, and can stimulate cancerogenic pathways [18]. Omega-3 polyunsaturated fatty acids suppress HMG-CoA reductase and have anticancerogenic properties through the induction of cell necrosis or apoptosis [19,20]. Other well-known anticancerogenic lipids that interfere with membrane-raft functions through cholesterol homeostasis are the alkylphospholipids, such as edelfosine [21]. George and Wu suggested that the significance of the membrane-raft composition and integrity for cell viability and proliferation is cell-type specific, due to fine tuning of the signaling pathways that can lead to cell death or cell survival [22]. Therefore, cholesterol-enriched membrane domains are potential targets for raft-disturbing agents, to affect cell proliferation and viability. Cytotoxic effects of such agents can lead to at least three forms of cell death: apoptosis, autophagic cell death, and necrosis [18,23,24]. Cholesterol depletion with methyl-β-cyclodextrin (MCD), which sequesters plasma-membrane cholesterol, renders melanoma cells susceptible to apoptosis [25] and triggers apoptosis in breast and prostate cancer cell lines, which have abundant membrane rafts [18].

In artificial and biological membranes, cholesterol/ sphingomyelin-rich membrane domains can be labeled with the ostreolysin A/ pleurotolysin B protein complex (OlyA/PlyB) both very selectively and with high affinity [26,27]. OlyA and PlyB are produced by the mushroom *Pleurotus ostreatus*, and they assemble into a pore-forming complex where each of the proteins has a particular role. OlyA serves as the membrane-binding component, and it binds exclusively to membrane domains that are enriched in sphingomyelin and sterols [26,27,28,29]. This binding can recruit PlyB to the membrane surface, leading to the formation of the 13-fold transmembrane pore, whereby each subunit is comprised of a PlyB molecule positioned on a membrane-bound OlyA dimer [26,30].

The interaction of OlyA with cholesterol/ sphingomyelin membranes is highly cooperative with respect to membrane cholesterol levels above an ~30 mol% threshold [28]. Once OlyA is recruited to these membranes, and if the concentration of OlyA/PlyB is high enough, PlyB has pore-forming activity [31]. The pre-treatment of cells with either MCD or sphingomyelinase dramatically abolishes the binding of OlyA [27] (and of OlyA/PlyB [3,32]) to the membranes of different cells.

To the best of our knowledge, there have been no reports on the role of membrane cholesterol as a potential target for the treatment of bladder cancer. In the present study, we investigated whether urothelial cells at different stages of cancerous transformation, and also nontransformed normal porcine urothelial (NPU) cells, differ in their sensitivities to cholesterol-interacting agents according to the differences in their cholesterol levels. To verify the influence of potential interspecies variability of cholesterol content, we measured the cholesterol content also in invasive and noninvasive mouse urothelial cell lines. We compared the sensitivity to MCD and OlyA/PlyB of T24 human urothelial cancer cells (as a high-grade invasive urothelial carcinoma model), RT4 human urothelial cancer cells (as a low-grade noninvasive papillary carcinoma model), and NPU cells, which morphologically and physiologically closely resemble normal human urothelium [33,34,35]. We took advantage of the unique mechanism of OlyA/PlyB protein complex to allow, on the one hand, efficient immunolabeling of cholesterol/ sphingomyelin-enriched membrane domains [3,32,36], and on the other hand, controlled pore-formation in these domains [28,29,32]. Our study demonstrates that these T24 and RT4 human urothelial cancer cells have markedly greater sensitivity for both cholesterol sequestration by MCD and pore-formation by OlyA/PlyB, compared to the nontransformed NPU cells. Moreover, these data offer a new and highly selective approach for treatment of life-threatening urothelial metastatic cells and the most-frequent noninvasive bladder cancer cells.

## Materials and Methods

### Cell cultures

The human urinary bladder T24 and RT4 cancer cell lines (ATTC, Manassas, VA, USA) and mouse urinary bladder, NUC-1 and g/G cells (a kind gift from Prof de Boer, Leiden University Medical Center, The Netherlands [37]) were cultured in advanced Dulbecco's modified Eagle's medium (A-DMEM)/F12 (1:1), 5% fetal calf serum (FCS), 100 U/ml penicillin, and 100 μg/ml streptomycin (control medium for cancer urothelial cells) at 37°C, in a humidified atmosphere with 5% CO_2_.

Secondary cultures of NPU cells from the fifth to twelfth passages were prepared as described previously [33,38,39]. The NPU cells were cultured in MCDB153 (Sigma-Aldrich, Taufkirchen, Germany)/A-DMEM (1:1), 2.5% FCS, 0.1 mM phosphoethanolamine (Sigma), 0.5 μg/ml hydrocortisone, 5 μg/ml insulin (Sigma), 4 mM glutamax, 100 U/ml penicillin, and 100 μg/ml streptomycin (control medium for normal urothelial cells). The culture media and supplements were purchased from Invitrogen (Vienna, Austria), unless otherwise stated.

### Methyl-β-cyclodextrin and OlyA/PlyB treatments

Methyl-β-cyclodextrin (MCD) was dissolved in control medium (for cancer or normal urothelial cells) using cholesterol-free FCS (Thermo Scientific Hyclone, Logan, UT, USA) instead of FCS with cholesterol.

A mixture of OlyA/PlyB (molar ratio 9:1) was prepared from fresh fruiting bodies of *P*. *ostreatus* as described previously [26,40]. Prior to the cell treatment, the OlyA/PlyB was diluted in cholesterol-free control medium (for cancer or normal urothelial cells). For membrane-raft labeling, a nonlytic concentration of OlyA/PlyB (2.5 μg/ml) was applied to the urothelial cells for 1 h and 3 h at 37°C. For studies of cytotoxity, OlyA/PlyB was used at 30 μg/ml for 1 h and 3 h at 37°C.

### Total cell cholesterol content

The T24, RT4, NUC-1 and g/G cells were seeded at 1 ×10^4^ cells/cm^2^, and the NPU cells at 1 ×10^5^ cells/cm^2^, and were grown in control medium to 100% confluence. The T24, RT4 and NPU cells were also treated with 7 mM MCD for 6 h, and 250-μl cell suspensions were prepared. The lipids were extracted from 200 μl of these cell suspensions, following the method of Bligh and Dyer [41]. These lipid extracts were dried with N_2_, and the lipids were dissolved in 50 μl isopropyl alcohol. Quantification of the free cholesterol in these lipid extracts was based on the use of cholesterol oxidase and the coupling of the hydrogen peroxide produced with 4-hydroxybenzoic acid and 4-aminopyridine with the peroxidase reaction. The quinoneimine dye that was formed as a result of the peroxidase was quantified at 560 nm (Konelab cholesterol kits, Thermo Fisher Scientific, Waltham, USA). Total protein concentrations were determined from 50 μl cell suspension aliquots using the Bradford assay [42]. The total cell cholesterol content is given as μg cholesterol/mg cell protein.

### OlyA and HMG-CoA reductase immunolabelings and fluorescence intensity measurements

The T24 and RT4 cells were cultured on coverslips and the NPU cells on 0.4-μm porous membranes (BD Falcon, Pharmingen, San Diego, CA, USA). For OlyA immunolabelling, cells were incubated with 2.5 μg/ml OlyA/PlyB for 30 min at 37°C. The cells were then washed with phosphate-buffered saline (PBS), and fixed in 4% formaldehyde (FA) for 10 min at 4°C. After 30 min of blocking with 2% bovine serum albumin (BSA) at 37°C, the cells were incubated with rabbit polyclonal anti- OlyA antibodies (1:2500) for 1 h at 37°C, then washed with PBS and incubated with Alexa Fluor 488-conjugated anti-rabbit secondary antibodies (1:600, Molecular Probes, Eugene, OR, USA) for 30 min at 37°C. For HMG-CoA reductase immunolabelling, cells were fixed in 4% FA for 10 min at 4°C, and put in blockade of 2% BSA at 37°C for 30 min. Then cells were incubated with rabbit anti- HMG-CoA reductase polyclonal antibodies (1:200, Merck Millipore 09–356) for 1 h at 37°C, washed with PBS and incubated with Alexa Fluor 488-conjugated anti-rabbit secondary antibodies for 30 min at 37°C.

The cells were mounted in Vectashield containing 4,6-diamidino-2-phenylindole (DAPI; Vector Laboratories, Burlingamme, CA, USA) and analyzed under fluorescent microscopy (AxioImager Z1) using an oil-immersion objective (63× oil/NA 1.40), and with an ApoTome device for optical section generation. The images were acquired using the AxioVision program (Carl Zeiss, Germany).

We analyzed 13–20 images per culture condition and measured the mean green fluorescence intensity per field of view (AxioVision program). The images were taken at the same exposure time (469 ms) for each cell culture condition.

### Immunoblotting analysis

After the MCD treatment, the cells were lysed with 2 mM EDTA, 150 mM NaCl, 100 mM Tris, 1% Triton X-100, 1 mM aprotinin, 1 mM phenylmethanesulfonylfluoride, and 1 mM Na_3_VO_4_, for 30 min at 4°C. Equal amounts of protein (20 μg) were resolved using SDS-PAGE and transferred to nitrocellulose membranes, which were then probed with rabbit anti-LC3 polyclonal antibodies (1:1000; MBL; PM036), rabbit anti-PARP polyclonal antibodies (1:1000; Roche Life Science; 11835238001), and rabbit anti-β-actin polyclonal antibodies (1:1000; Sigma; A2066). Horse-radish-peroxidase-conjugated anti-rabbit polyclonal secondary antibodies (1:1000; Sigma) were detected using the enhanced chemiluminescence technique (Pierce ECL Western blotting substrate, Thermo Scientific).

### Measurement of caspase activity

The caspase activity was determined by measuring cleavage of acetyl-Asp-Glu-Val-Asp-7-amino-4-trifluoromethylcoumarin (Ac-DEVD-AFC; Bachem, Bubendorf, Switzerland) in nontreated (control) and MCD-treated T24, RT4, and NPU cells as described previously [43]. For the positive control of caspase activation, RT4 cells were exposed to UV irradiation for 30 s to induce apoptosis, and then incubated for additional 6 h. The DEVD-ase activity was measured using a microplate reader (Safire; Tecan, Mannedorf, Switzerland). The initial rates of the reactions were calculated and are presented as relative fluorescent units per seconds (RFU/s).

### Flow cytometry

The treated and nontreated urothelial cells were cultured in 24-well plates (1 ×10^5^ cells/well) and grown to confluence for the MCD and OlyA/PlyB treatments. The harvested cells were incubated with the annexin V–PE reagent (BD Biosciences, Pharmingen, San Diego, CA, USA) and with 0.2 μg/ml propidium iodide (PI; Sigma).The cells were analyzed for annexin V–PE and PI fluorescence with a FACSCalibur flow cytometer and CellQuest software (both Becton Dickinson San Jose, CA, USA). We discriminated the fractions of viable (annexin V negative and PI negative), early apoptotic (annexin V positive, PI negative), and dead (PI positive) cell populations. Three independent experiments were performed, with each carried out in duplicate.

### Transmission electron microscopy

The cells treated with MCD and OlyA/PlyB and the untreated cells were fixed with 2.5% glutaraldehyde in cacodylate buffer for 3 h at 4°C. After washing, the cells were incubated with 1% OsO_4_ and 3% K_4_Fe(CN)_6_, prepared in cacodylate buffer, for 1 h at room temperature. Then 0.3% thiocarbohydrozide in cacodylate buffer was added for 5 min at room temperature. After washing, 1% OsO_4_ was added for 20 min at room temperature. The cells were then dehydrated and embedded in Epon (Serva, Heidelberg, Germany). Ultrathin sections were stained with uranyl acetate and lead citrate (both from Merck, Darmstadt, Germany). These sections were examined under transmission electron microscopy (TEM) using a Phillips CM100 electron microscope.

### Statistics

The data are presented as means ±standard error of two or three independent experiments, each performed in duplicate or triplicate, and were evaluated using Students’ t-tests, and one-way ANOVA or Kruskal-Wallis one way analysis of variance. Where pairwise multiple comparisons were needed, the Holm-Sidak method or the Dunn's method were used (Sigma Plot software, Systat Software Inc, CA, USA). P-values <0.05 were considered to be statistically significant. The Pearson's correlation coefficient between cholesterol content and HMG-CoA reductase protein expression was calculated (Microsoft Office Excel).

## Results

### Cholesterol content is higher in invasive urothelial cancer cells in comparison to noninvasive urothelial cells of human and mouse origin

**Fig 1:**
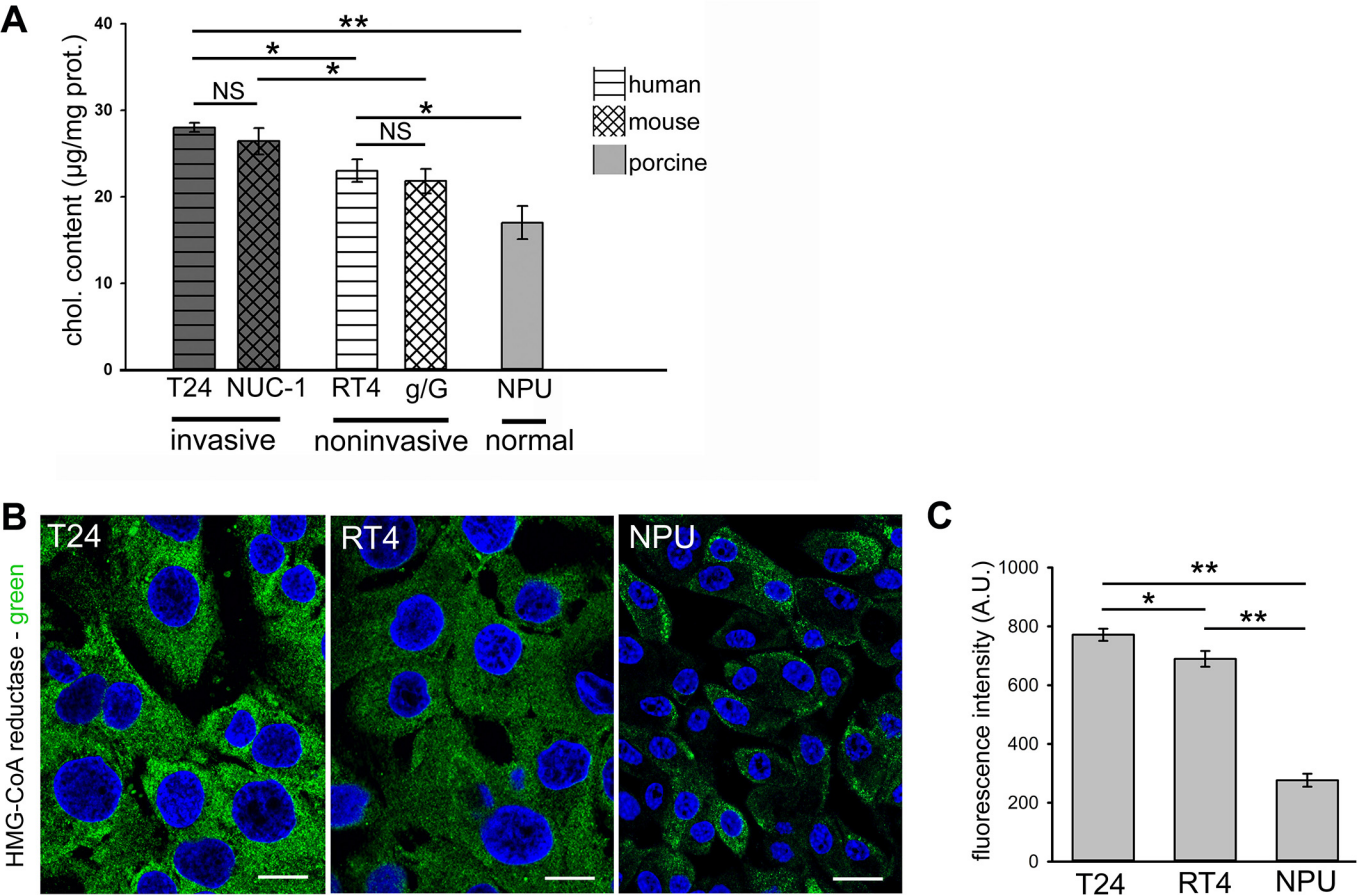
Cholesterol content and HMG-CoA reductase distribution in urothelial cells. **A.** Quantification of cholesterol content in invasive T24 human and NUC-1 mouse cells, in noninvasive RT4 human and g/G mouse cells, and in NPU cells. **B.** Representative optical sections of HMG-CoA reductase immunolabeling (green) in T24, RT4 and NPU cells. DAPI nuclear staining is also seen (blue). Scale bars 20 μm. **C.** Quantification of the mean intensity of HMG-CoA immunofluorescence of T24, RT4 human cells and NPU cells, as illustrated in (B). **A, C.** Data are means ±standard errors of three independent experiments. NS, not significant; *p <0.05, **p <0.005.

For the human urothelial cells, analysis of the cholesterol contents revealed the highest levels in T24 invasive urothelial cancer cells, which were significantly lower in RT4 noninvasive urothelial cancer cells, and lowest in NPU cells (28.0 ±0.51; 22.9 ±1.3; 16.6 ±1.9 μg cholesterol/mg cell protein, respectively; Fig 1A). There was a positive correlation between cholesterol content and HMG CoA reductase protein expression, calculated as Pearson's correlation coefficient being r = 0.945 measured from the mean intensity of the immunofluorescence (Fig 1B). This immunofluorescence was the highest in T24 cells and lowest in NPU cells.

As these nontransformed NPU cells are of porcine origin, to evaluate any interspecies differences in cholesterol content, we analyzed cholesterol content of invasive and noninvasive urothelial cells of mouse origin. Here, we tested NUC-1 and g/G urothelial cells, that reflect the distinct phases in urothelial cancerogenesis in mice [37]. NUC-1 cells are tumorigenic and invasive, and as such, they provide a close comparison to T24 human cells, and g/G mouse urothelial cells are noninvasive, and are thus comparable to RT4 human cells. The cholesterol content in the invasive NUC-1 cells was significantly higher than in the noninvasive g/G cells (26.3 ±1.5; 21.8 ±1.4 μg cholesterol/mg cell protein, respectively; Fig 1A). On the other hand, the cholesterol content of T24 human and NUC-1 mouse invasive urothelial cells did not differ significantly, as also seen for the cholesterol content in RT4 human and g/G mouse noninvasive urothelial cells. These data indicate that the content of cholesterol has a reverse correlation with the level of urothelial cancer transformation (Fig 1A).

### MCD induces time-dependent and dose-dependent death of urothelial cancer cells

**Fig 2:**
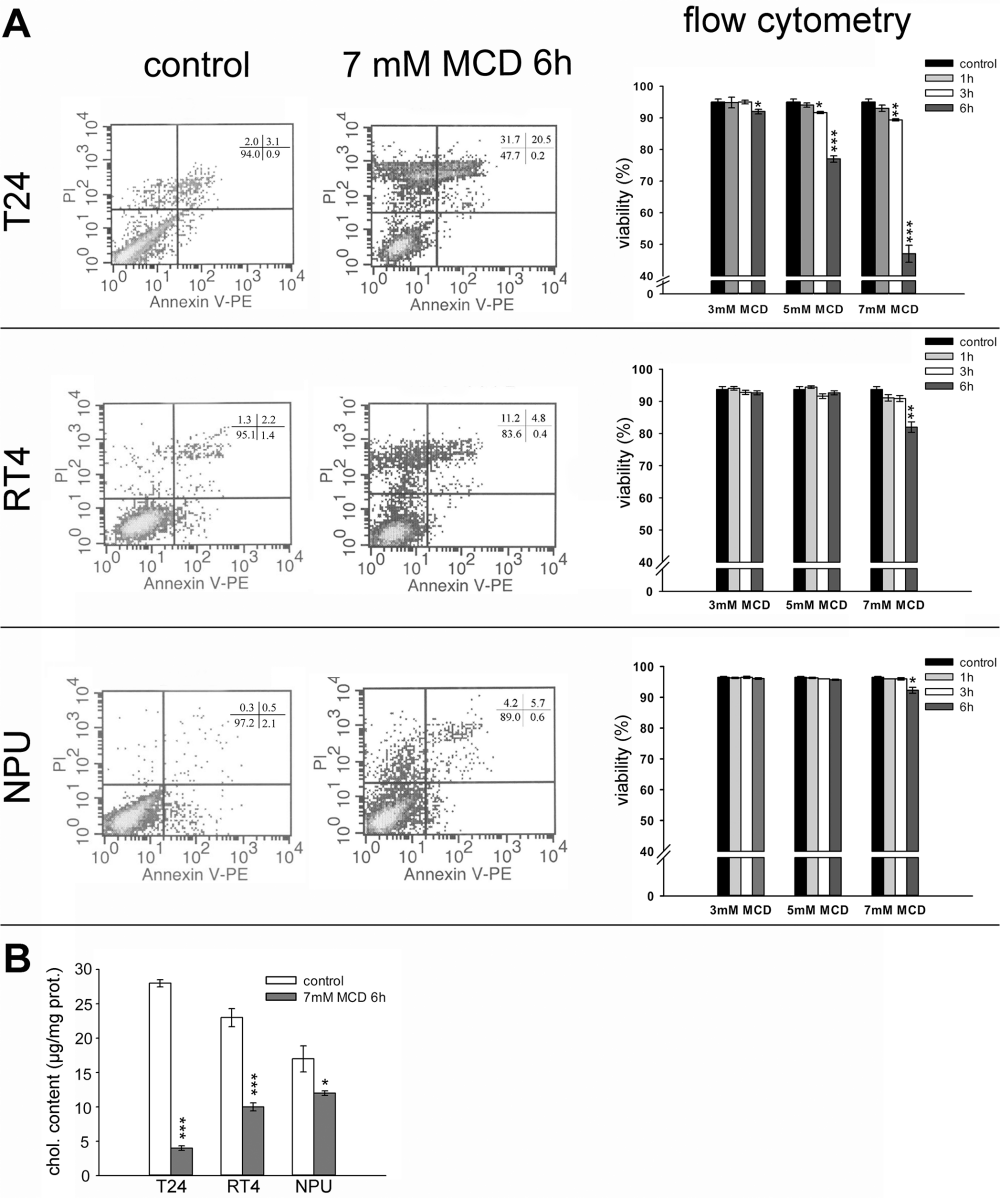
Cell viability and cholesterol content of urothelial cells after MCD treatments. **A.** Left: Representative dot plots from flow cytometry analysis of T24, RT4 and NPU cells grown in control media and after 7 mM MCD treatment for 6 h. Annexin V–PE and PI were used to discriminate between viable (double negative), early apoptotic (single annexin V positive) and dead (PI positive) cells. Right: Quantification of the cell viability from the flow cytometry analysis of T24, RT4 and NPU cells after 3 mM, 5 mM, and 7 mM MCD treatments for 1 h, 3 h and 6 h. **B.** Quantification of cell cholesterol depletion in T24, RT4 and NPU cells after 7 mM MCD treatment for 6 h. Data are means ±standard errors of duplicate measurements from three independent experiments. *p <0.05, **p <0.005, ***p <0.001.

The effect of MCD treatment on cell viability was investigated using annexin V and PI staining in combination with flow cytometry analysis. The representative dot plots in Fig 2A show T24, RT4 and NPU cell samples treated with 7 mM MCD for 6 h, and these are accompanied by quantification of the effects at different concentrations of MCD (3, 5, 7 mM) applied for increasing incubation times (1, 3, 6 h). At 3 mM MCD, no or only minor effects on the viabilities were observed in all three cell types, regardless of the incubation time (Fig 2A).

In T24 cells, which were the most sensitive to MCD of these three cell types, cell viability was significantly reduced after 5 mM and 7 mM MCD treatment for 3 h, to 92% and 89%, respectively. Six hours after the treatment viability was further reduced to 77% at 5 mM MCD and to only 47% at 7 mM MCD (Fig 2A). In RT4 cells, the only significant reduction in viability over a range of MCD concentrations and incubation times was after 6 h with 7 mM MCD (to 82%; Fig 2A). An even smaller effect was observed for NPU cells, where only the treatment with 7 mM MCD for 6 h showed a minor reduction in cell viability (to 92%; Fig 2A). When compared to T24 and RT4 cells, the effect of 6-h treatment with 7 mM MCD on cell viability was statistically lower in NPU cells (Fig 2A). In addition, we could observe that dying, PI positive cells were annexin V negative or positive, whereas a population of single annexin V positive cells was never prominent, which suggests that the cell death is necrotic rather than apoptotic (Fig 2A).

Interestingly, after 7 mM MCD treatment for 6 h, the total cholesterol content of T24, RT4, and NPU cells was reduced in comparison to the respective untreated cells, by 86%, 57%, and 29%, respectively (Fig 2B).

**Fig 3:**
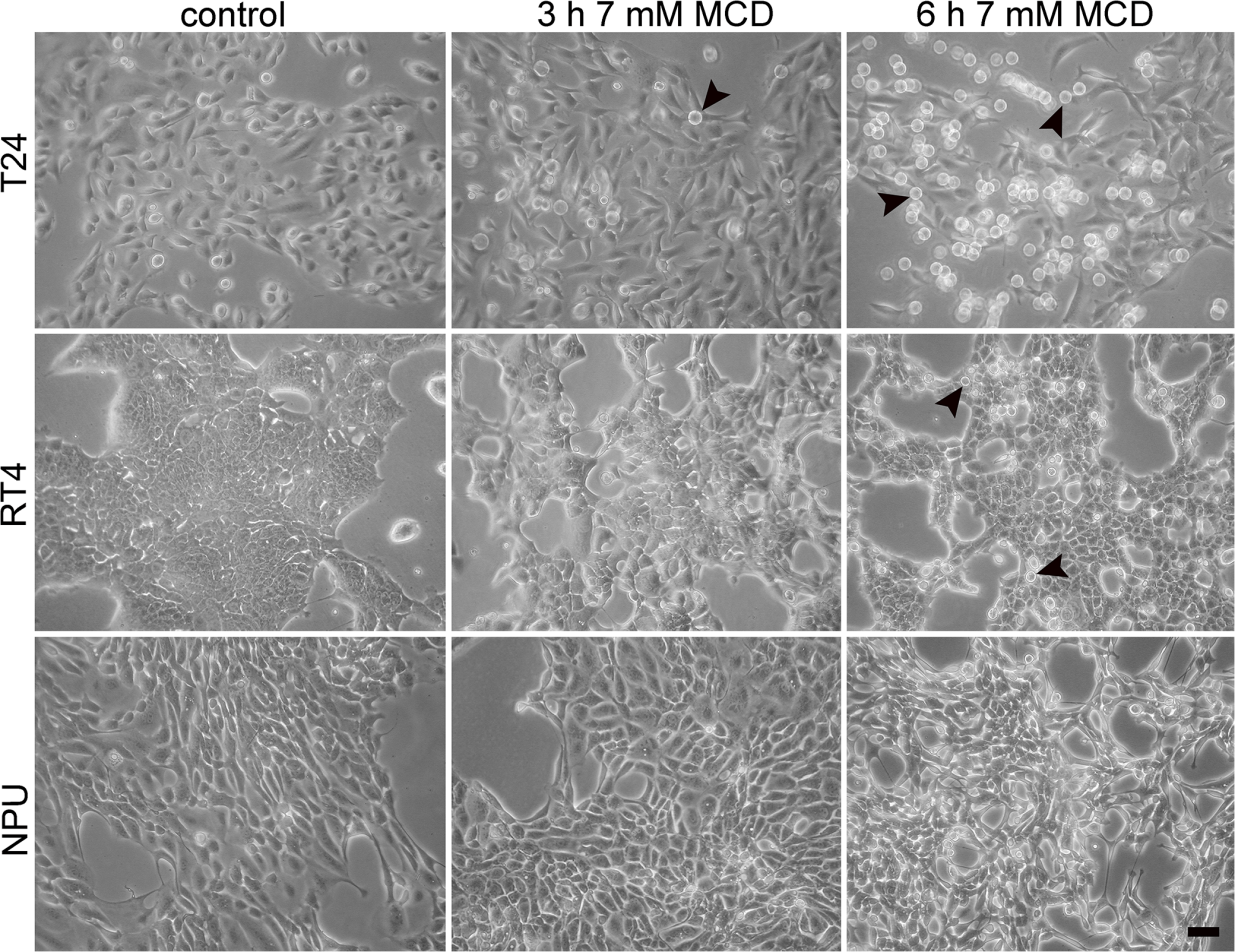
Morphological changes of urothelial cells after treatment with 7 mM MCD. T24, RT4 and NPU cells (as indicated) were untreated (control) or treated with 7 mM MCD for 3 h and 6 h, and then examined under an inverted microscope. Cell rounding was cell-type dependent (T24 > RT4 > NPU) and time dependent (control < 3 h < 6 h). T24 and RT4 cells changed shape from flat and polygonal (control) to spherical (T24 cells, 3 h, 6 h treatment; RT4 cells, 6 h treatment; arrowheads). Scale bar, 20 μm.

The analysis of cell viability thus showed that the extent of cytotoxicity of MCD increased with the concentration and duration of the treatment. To further define these different sensitivities to MCD treatment of these three types of urothelial cells, cell morphology was analyzed after 7 mM MCD treatment, where significantly decreases in cell viability were seen. T24 cells treated with 7 mM MCD for 3 h, and RT4 cells treated with 7 mM MCD for 6 h, became rounded and were weakly attached (Fig 3). T24 cells detached after 7 mM MCD treatment for 6 h (Fig 3). The extent of cell rounding was most pronounced in T24 cells, and less pronounced in RT4 and NPU cells, where only minor morphological effects were observed. These data correlated well with the cell-death analysis.

**Fig 4:**
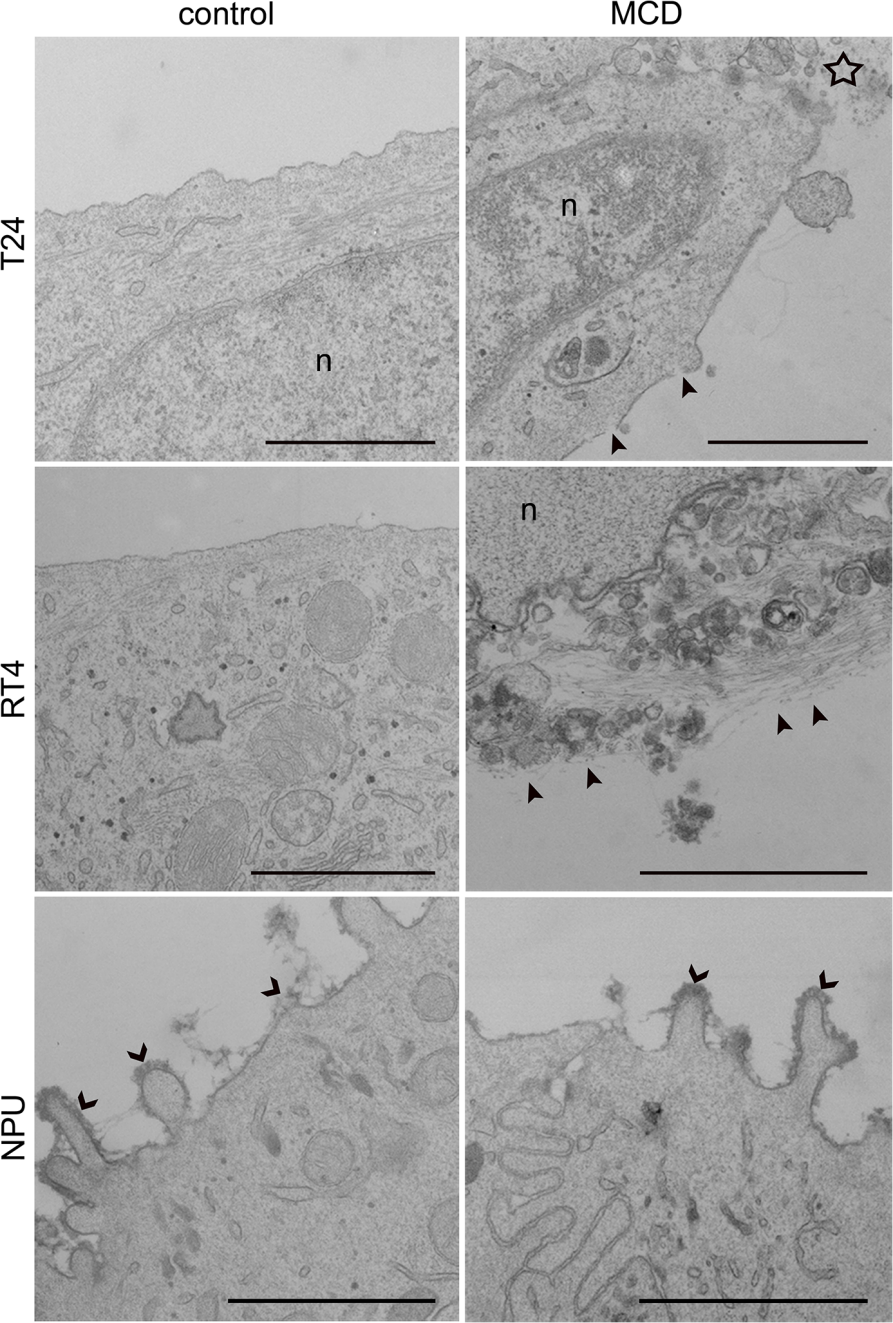
Ultrastructure of urothelial cells after treatment with 7 mM MCD. T24, RT4 and NPU cells were untreated (control) or treated with 7 mM MCD for 6 h, and then processed for TEM. Necrotic changes were seen in individual T24 cells (star) and RT4 cells, including plasma-membrane disruption (arrowheads) and release of cell contents. NPU cells remained intact after this MCD treatment, with glycocalyx still present (open arrowheads). n, nucleus. Scale bars, 1 μm.

In all cases, with the PI-positive staining indicating that cell viability was reduced due to necrosis, ultrastructural analysis by TEM provided further evidence of necrotic cell death (Fig 4). The plasma membranes of T24 and RT4 cells after 7 mM MCD treatment for 6 h were ruptured and the cytoplasm was released, although the nuclear envelope remained intact (Fig 4). However, NPU cells showed no characteristics of necrosis after this MCD treatment (Fig 4).

**Fig 5:**
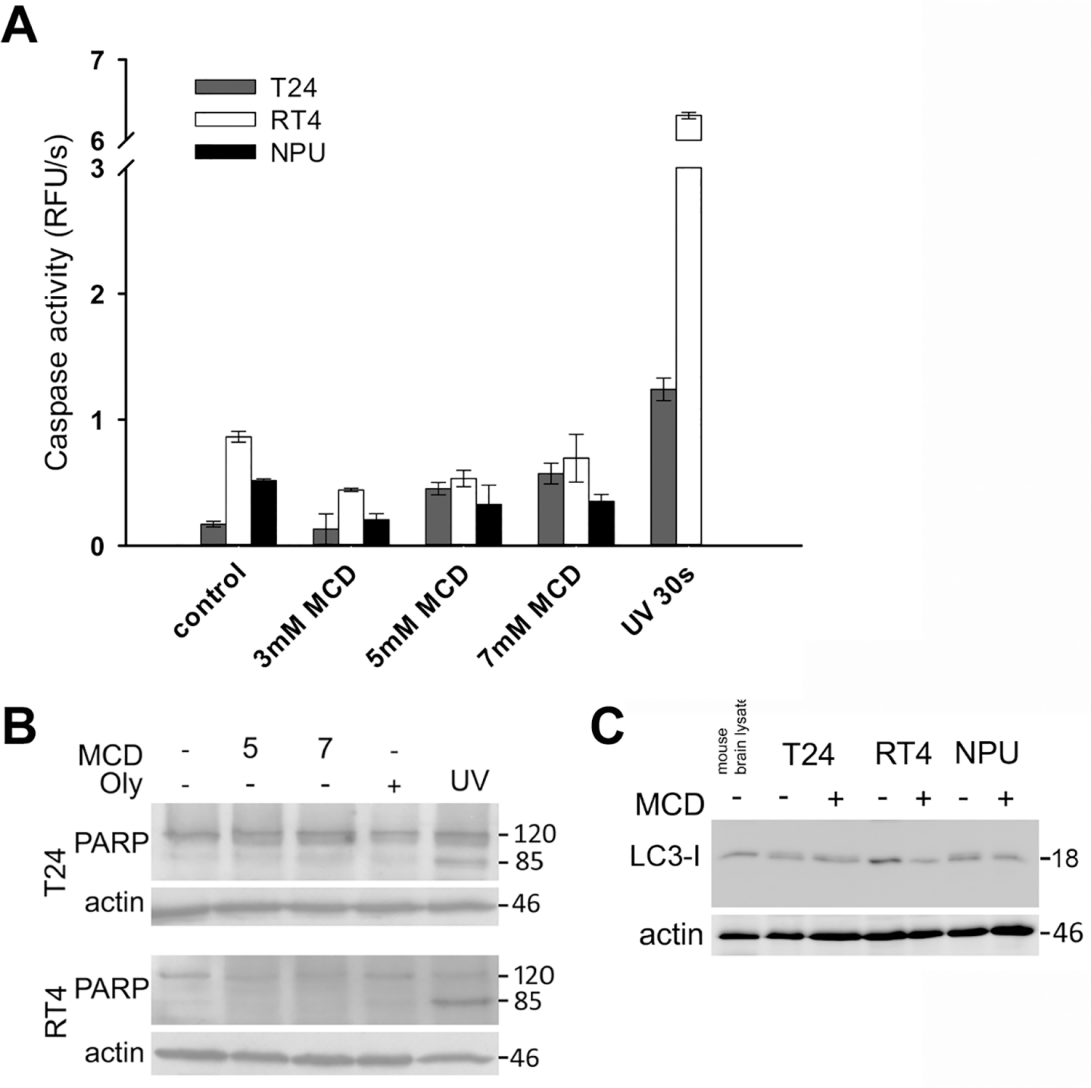
No apoptosis or autophagy of urothelial cells after MCD treatments for 6 h. T24, RT4 and NPU cells were treated with 3 mM (A), 5 mM (A) or 7 mM (A-C) MCD for 6 h. **A.** Caspase activity measurements by Ac-DEVD-AFC cleavage did not show any increase in caspase activity. The positive controls (UV 30s) showed apoptosis-related caspase activities induced in T24 and RT4 cells at 6 h. **B.** Immunoblotting analysis did not show cleavage of full-length PARP (120 kDa) into an 85-kDa fragment. **C.** Immunoblotting analysis did not show conversion of LC3-I (18 kDa) to LC3-II (16 kDa). Actin was used as the loading control. Data are means ±standard error of triplicate measurements.

To more specifically investigate involvement of apoptosis after MCD treatment, the activation of caspases was analyzed. By monitoring the cleavage of the fluorogenic Ac-DEVD-AFC substrate no significant caspase activation was observed upon 6 h of incubation with 3 mM, 5 mM or 7 mM MCD either in T24, RT4 or NPU cells. (Fig 5A). We were also unable to detect caspase activation in cells incubated with 5 and 7mM MCD for 6 hours by immunoblotting of the PARP p85 fragment (Fig 5B), which is produced by activated caspases [44]. For comparison, UV radiation caused the cleavage of both the DEVD peptide and the PARP protein in T24 and RT4 cells. The lack of caspase activation combined with the absence of annexin V signal and ultrastructural changes characteristic of necrosis collectively suggested that the MCD treatment of urothelial cells triggered necrotic rather than apoptotic cell death.

Western blotting of T24, RT4 and NPU cells after treatment with 7 mM MCD for 6 h did not reveal any conversion of LC3-I to LC3-II (Fig 5C). This indicated that MCD did not induce autophagy. Moreover, observation of T24, RT4 and NPU cells by TEM did not reveal any typical autophagic structures, such as double-membrane vesicles or autophagosomes (Fig 4).

### OlyA/PlyB-treatment induces necrosis of urothelial cancer cells

**Fig 6:**
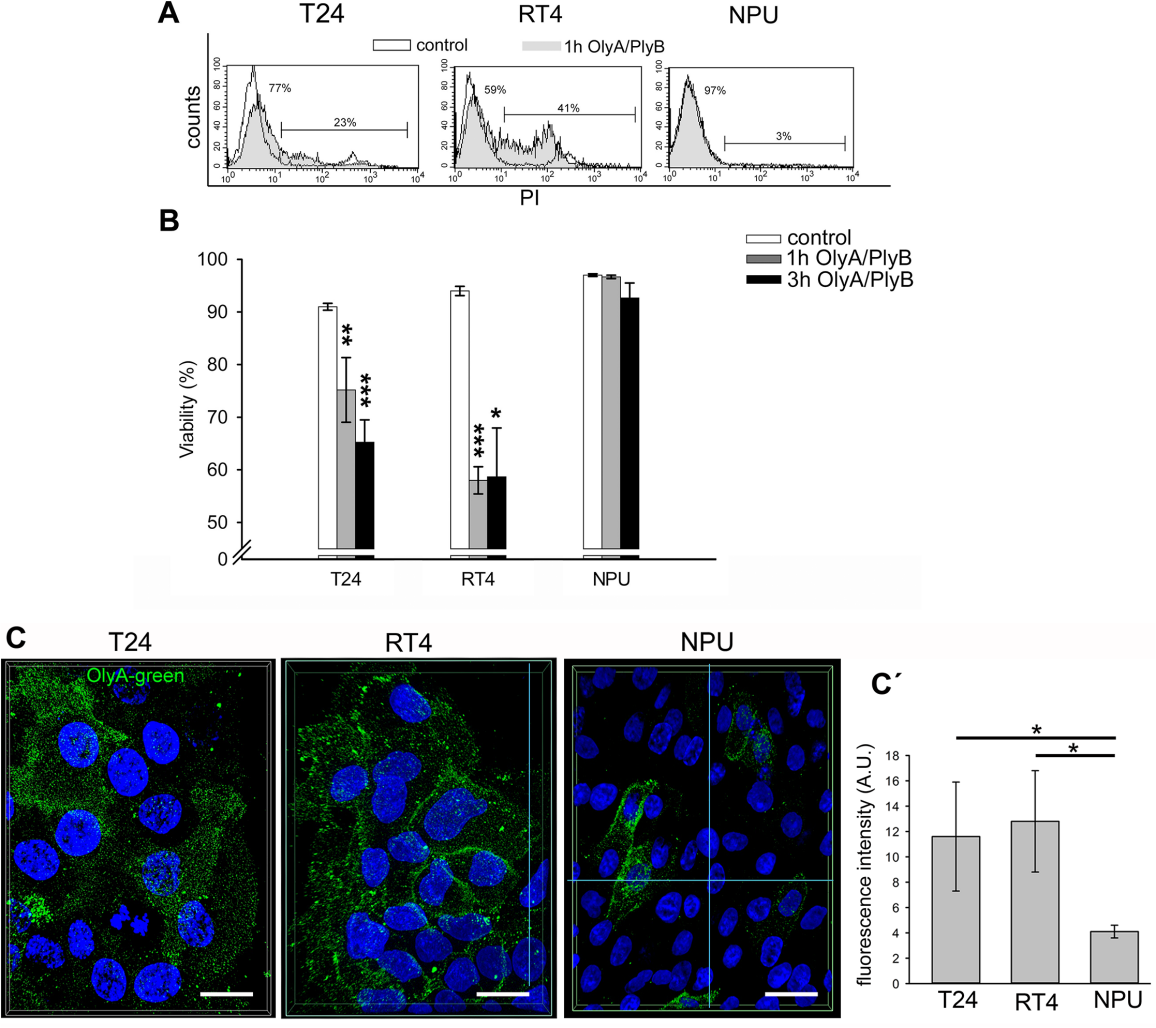
Viability of urothelial cells after OlyA/PlyB treatment. **A.** Representative distribution of PI staining of T24, RT4 and NPU cells treated with 30 μg/ml Oly/PlyB for 1 h. Open histograms represent untreated cells, while filled histograms denote OlyA/PlyB-treated cells. The percentages of live and dead cells are shown on the left and right part of the histograms, respectively. **B.** Viability of T24, RT4 and NPU cells following 1-h and 3-h treatments with 30 μg/ml OlyA/PlyB, as determined by flow cytometry analysis. Data are means ± SE of duplicate measurements from two independent experiments. **C**. Immunolabeling of cholesterol/sphingomyelin rich membrane domains in the urothelial cells with OlyA. The superimposed image of optical sections through entire cells represent the extent and the distribution of OlyA immunolabeled cholesterol/ sphingomyelin rich membrane domains in T24, RT4 and NPU cells. Green, OlyA-labeling; blue, DAPI staining of nuclei. Scale bars, 20 μm. **C΄**. The quantification of OlyA immunolabeling is presented as the mean fluorescence intensity per field of view (in arbitrary units; A.U.). *p <0.05, **p <0.005, ***p <0.001.

OlyA/PlyB was used for two different applications. A nonlytic concentration of OlyA/PlyB (2.5 μg/ml) was used to label cholesterol/ sphingomyelin-rich domains of the plasma membranes (Fig 6C), and a lytic concentration (30 μg/ml) was applied to permeabilize plasma membranes. The most prominent OlyA immunolabeling (antibodies were raised against OlyA respectively) was seen for RT4 cells and revealed by quantification of OlyA fluorescence (Fig 6C'). This OlyA labeling was more uniform over the plasma membrane in RT4 cells. In contrast, the OlyA distribution in T24 cells was less even, and its distribution was discontinuous. Labeling of NPU cells showed fewer OlyA-positive cells that significantly differs from T24 and RT4 cells (Fig 6C).

Cell viability after 1-h and 3-h treatments with 30 μg/ml OlyA/PlyB was analyzed by flow cytometry. The cell viabilities after 30 μg/ml OlyA/PlyB treatments for 1 h and 3 h were determined as above, by flow cytometry analysis. As the annexin-V labeling was negative in all of the cells, the fractions of the PI-positive (i.e., dead) and PI-negative cells were determined as indicated by the representative histograms shown in Fig 6A. After 30 μg/ml OlyA/PlyB treatment for 1 h, the cell viability of T24 cells was significantly decreased to 75% (p <0.005), and after 3-h, to 65% (p <0.001). Similarly, the cell viability of RT4 cells decreased to 58% (p <0.001) after 1 h, although this did not change further for the 3-h treatment. In contrast, the cell viability of NPU cells was not significantly affected by the 30 μg/ml OlyA/PlyB treatments for either 1 h or 3 h (Fig 6B). Across all of these three cell types tested, the differences in the cell viabilities between the 30 μg/ml OlyA/PlyB treatments for 1 h and 3 h did not reach significance. The results of immunolabeling of cholesterol/sphingomyelin rich membrane domains with OlyA were in accordance with the analysis of cell viability.

**Fig 7:**
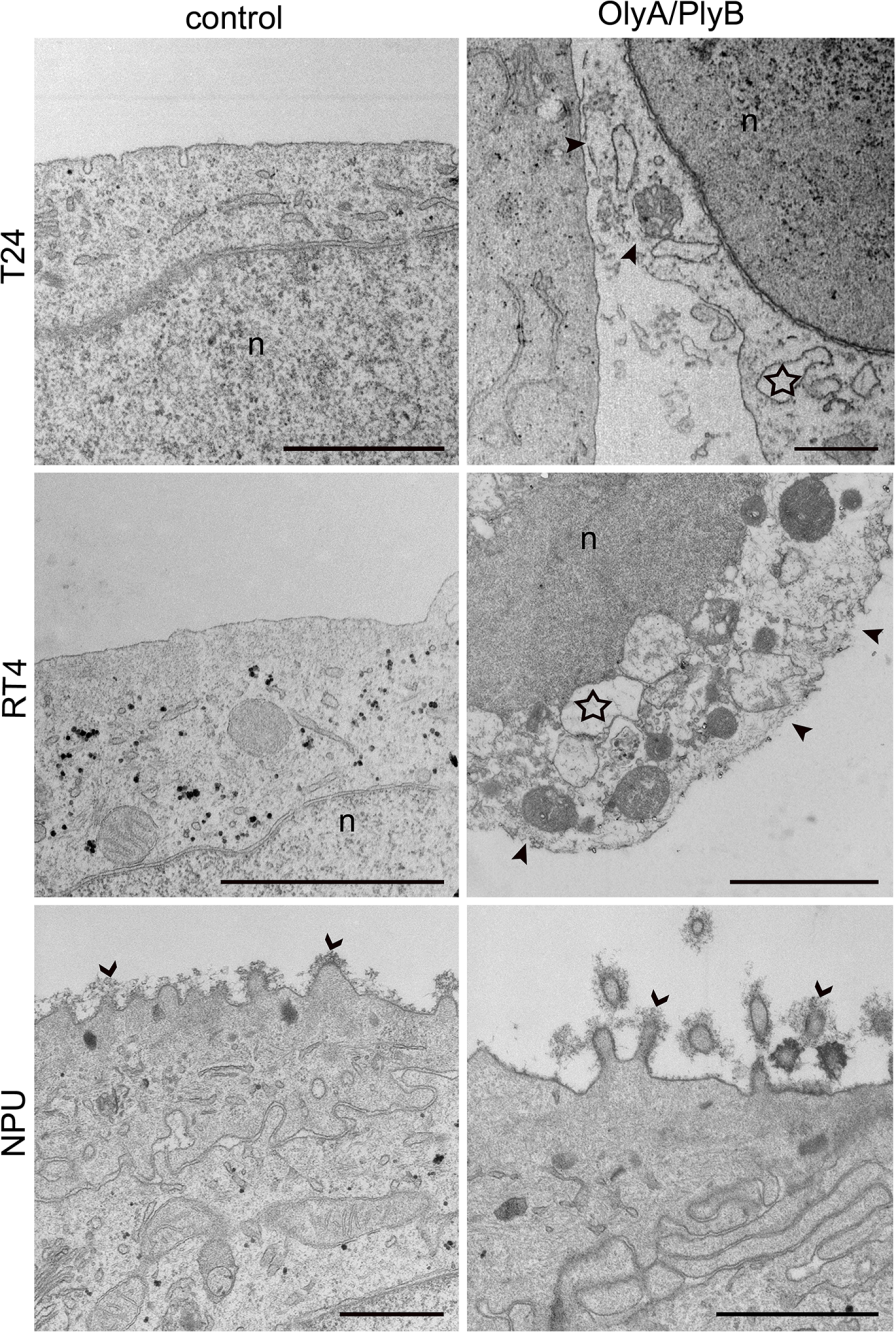
Ultrastructure of urothelial cells after treatment with 30 μg/ml OlyA/PlyB. T24, RT4 and NPU cells were treated with 30 μg/ml OlyA/PlyB for 1 h and then processed for TEM. RT4 and T24 cells show loss of membrane integrity (arrowheads), release of cytoplasm, and organelle swelling (stars), which indicate cell necrosis. The ultrastructure of treated NPU cells resembles that of untreated NPU cells, with abundant glycocalyx (open arrowheads). n, nucleus. Scale bars, 1 μm.

Necrotic cell death of T24 and RT4 cells after 30 μg/ml OlyA/PlyB treatment for 1 h was indicated by the PI-positive cells (Fig 6A) and their characteristic ultrastructural changes (Fig 7). For these cells, the most prominent changes were seen as increased plasma-membrane permeability and leakage of cytoplasm, along with organelle swelling (Fig 7). The ultrastructure of NPU cells was not affected by 30 μg/ml OlyA/PlyB treatment for 1 h, with these control and treated cells showing similar morphology and with intact plasma membrane (Fig 7).

## Discussion

Bladder cancer is the fourth most common cancer diagnosis in men [45]. However, because of the high recurrence rate and the need for ongoing invasive monitoring, it has the highest lifetime treatment costs per patient of all cancers [46]. Indeed, up to 70% of patients have local recurrences after intravesical chemotherapy or immunotherapy [47]. It is likely that the low cure rate is due to micrometastatic disease that can be induced at the time of transurethral resection or cystectomy, which results in relapse of the urothelial tumors or in metastases in the lymph nodes, bones, lung, skin, and liver [48]. Moreover, mortality is mainly caused by invasive, metastatic urothelial carcinomas that become resistant to chemotherapy. For this reason, new anticancer therapies involving different approaches are being investigated to treat bladder cancer.

In the present study, the characteristic differences in the cholesterol content between NPU cells and cancerous urothelial cells was initially established and then further used to define selective targeting in the treatment of these cancer cells. The lipid composition of the cell membranes has been shown to change early in carcinogenesis, and it is severely affected when normal cells are transformed into malignant cells in breast cancer [49], which suggests that membrane lipid composition has an important role in cancer progression. Specifically, the composition and dynamics of membrane rafts and their involvement in cell signaling appear to be pivotal in cancer development [17].

In this respect, however, no similar data are available for bladder cancer. Thus, two human urothelial cell lines with different levels of cancer transformation, as T24 and RT4 cells, were analyzed for cytotoxicity along with NPU cells in the present study. Since normal human urothelium is difficult-to-obtain tissue, we used normal porcine urothelial cell culture, which shows identical differentiation markers as well as cell biological and histological similarities to human urothelium [35,50].

To this end, we show here that cholesterol content increases in correlation with the transformation grade, from nontransformed NPU cells, over RT4 low-grade human papillary cancer cells to T24 high-grade invasive human urothelial cancer cells,. Among the possible mechanisms that provoke up-regulation of cellular cholesterol synthesis there is increased activity of HMG-CoA reductase. Our quantification of HMG-CoA reductase protein expression clearly corresponded to the analyses of the cholesterol concentrations in these cells. We additionally show that the same correlation is evident for similar cancer transformation in mouse cells. Cholesterol content in invasive NUC-1 mouse cells was higher than in non-tumorigenic g/G mouse cells, providing strong evidence that elevated cholesterol content is a characteristic of high-grade urothelial cancer. It is likely that the increase in cholesterol content in T24 and NUC-1 cells originates from high cholesterol import and up-regulated cholesterol synthesis, as has been shown in other malignancies [51,52,53]. These increased cholesterol levels might serve for biogenesis of new membranes during the accelerated proliferation of these cancer cells.

Our aim was to determine whether MCD and OlyA/PlyB affect cell viability of T24 and RT4 urothelial cancer cells compared to NPU cells, and whether this is related to different cholesterol contents of urothelial cells. Presented data show that MCD treatment reduces viability of these urothelial cancer cells in time-dependent and dose-dependent manners. There was significant reduction in cell viability of T24 cells, which is in agreement with their highest content of cholesterol and which would mean that cholesterol is more available to MCD in this cell type. DEVD-ase activity and ultrastructure analyses revealed that MCD treatment of T24 and RT4 urothelial cancer cells induced necrosis rather than apoptosis, thereby excluding the classical apoptotic pathway. This is consistent with previous studies suggesting that necrotic cell death can be the consequence of alterations to the membrane fluidity and/or breakdown of cell membrane integrity [6,54]. This is also in agreement with previous reports for breast cancer, where lipid composition was shown to be of major importance in cancer progression [49]. The cholesterol-depletion in the present study clearly shows that the availability of cholesterol and their sensitivity to MCD treatment is increased in high-grade urothelial cells. On the other hand, NPU cells, which had the lowest cholesterol content, were the most resistant to the MCD extraction. This can be interpreted in terms of the chemical activity or availability of cholesterol in the membranes, as related to its cellular distribution and homeostasis. It has been shown recently that the availability of cholesterol for both MCD and cholesterol oxidase increases above a cholesterol content threshold that is specific for each cholesterol/ phospholipid mixture and for red blood cell membranes [2]. One possible explanation for NPU resistance in comparison to cancer cells could be that in differentiated urothelial cells apical plasma membranes contain special proteins uroplakins which arrange urothelial plaques [55] as crystal structures and give urothelial plasma membranes rigidity and inaccessibility to MCD. Thus, our data suggest that the level of cellular cholesterol can be considered as an indicator of the malignancy of urothelial cells and the efficiency of its depletion with MCD can potentially be used for selective removal of invasive urothelial cancer cells.

Lytic OlyA/PlyB treatment caused the greatest decrease in viability of RT4 cells, and less of T24 cells, while it had no effect on the viability of nontransformed NPU cells. This correlates well with the extent of OlyA localization and quantification to cholesterol/ sphingomyelin-rich membrane domains in these cells, as determined with OlyA immunolabeling. These data indicate diversity in the abundance of the cholesterol/ sphingomyelin membrane domains among these urothelial cells at different stages of cancer transformation, and in comparison with the nontransformed NPU cells. Indeed, it was shown that determination of the membrane lipid profile can distinguish between different phases of cancer development [56].

Analyses of cell viability and ultrastructure revealed that OlyA/PlyB-induced cell death is not apoptotic, but necrotic, similar to the MCD treatment. Necrosis after OlyA/PlyB treatment was shown here to be the prevalent cell death mechanism for RT4 cells, and was most likely caused by membrane perforation. It has been reported previously that the interactions of OlyA, as well as with the OlyA/PlyB protein mixture, with cholesterol/ sphingomyelin membranes is highly cooperative with respect to membrane cholesterol concentrations above a ~30 mol% threshold [28,29,32].

Our data thus indicate that cholesterol-rich membrane domains provide a new target for treatment of bladder cancer. *In vivo*, either MCD or OlyA/PlyB might be applied transureterally into the bladder cavity as supportive reagents for the elimination of micrometastases (i.e., considering T24 cells as a model) or against recurring papilloma cells (i.e., considering RT4 cells as a model). As shown in our model system, NPU cells should not be particularly harmed by this treatment, as neither MCD nor OlyA/PlyB affected the viability of NPU cells. Of course, these analyses of the cholesterol content and the responses to MCD and OlyA/PlyB will need to be confirmed also with a model of well-differentiated human urothelial cells. Unfortunately, such *in-vitro* model is very difficult to obtain, because of the limited sources of healthy urothelial tissue and the need for constant renewal of the cultured cells, which can otherwise not achieve highly differentiated levels.

From our previous analyses, we can conclude that the potential minor damage caused by application of MCD and OlyA/PlyB would not be detrimental to normal urothelial tissue, because of the high regenerative capacity of these cells under *in-vivo* conditions [57]. What is more, MCD or OlyA/PlyB-mediated release of the intracellular contents from necrotic cancer cells might represent an immunomodulatory event, and might initiate a targeted and efficient immune response, as in Bacillus Calmette–Guérin-treated patients with bladder carcinoma [24].

The experimental data from the present study on MCD and OlyA/PlyB demonstrate that both of these cholesterol-disturbing agents can selectively decrease the viability of urothelial cancer cells. However, although urothelial cancer cell lines are invaluable in studies of cancer-cell behavior, such *in-vitro* studies neglect the important control of cell growth and differentiation of the *in-vivo* tissue environment. To confirm the therapeutic potential of MCD and OlyA/PlyB, both ofagents need to be evaluated in biomimetic *in-vitro* models or in animal models with orthotopic bladder tumors.

## Conclusions

To summarize, we have demonstrated that in these urothelial cancer cells, both the increased content of cholesterol and the increased levels of cholesterol/ sphingomyelin-rich membrane domains constitute therapeutic targets for selective induction of cell death. MCD was more toxic to the metastatic T24 high-grade urothelial cancer cells, and OlyA/PlyB to the RT4 low-grade urothelial cancer cells. Both MCD and OlyA/PlyB leave the nontransformed NPU cells largely intact. Therefore, this study is aimed to stimulate further research into the area of lipid-related targeting that might provide new locally applied therapies that can be used for elimination of urothelial cancer cells, with the prospect of restricting tumorigenesis and urothelial tumor recurrence.
